# The complete chloroplast genome of the monotypic genus of *Bulleyia* Schltr. (Orchidaceae)

**DOI:** 10.1080/23802359.2020.1831983

**Published:** 2020-11-03

**Authors:** Jing Ai, Yan-Ping Wang, Hui-Jun Guo, Lu Li

**Affiliations:** a Department of Biodiversity Conservation, Southwest Forestry University, Kunming, China; b Department of Life Science, Southwest Forestry University, Kunming, China; c Southwest Forestry University, Kunming, China

**Keywords:** *Bulleyia yunnanensis*, chloroplast genome, phylogenetic analysis, Orchidaceae

## Abstract

*Bulleyia* Schltr. is monotypic and represented only by *Bulleyia yunnanensis* Schltr., native to the Yunnan of China, Bhutan, and northeast India. Here, we report the complete chloroplast (cp) genome sequence and the cp genome features of *B. yunnanensis*. The cp genome sequence of *B. yunnanensis* was 159,581 bp in length and presented a typical quadripartite structure consisting of one large single-copy region (LSC, 87,563 bp), one small single-copy region (SSC, 18,714 bp), and two inverted repeat regions (IR, 26,652 bp). Besides, the cp genome encoded 132 genes, including 113 unique genes (79 protein-coding genes, 30 tRNA genes, and four rRNA genes). The phylogenetic analysis suggested that *B. yunnanensis* be closely related to *Pholidota* in tribe Arethuseae.

*Bulleyia yunnanensis* is a perennial epiphytic orchid with high medicinal value (Lu and Zhang 2015; Wang et al. 2015), this species is characterized by compressed pseudobulbs, pendulous racemose with 10–20 white, small and distichous flowers (Chen et al. 2009). According to the recent classification of Orchidaceae, *Bulleyia* was placed in tribe Arethuseae Lindl. of subfamily Epidendroideae (Orchidaceae), in which tribal relationships were unclear (Li et al. 2016). Since the plastid genome would play a key role in plant systematics and evolution (Jakobsson et al. 2007), the complete cp genome of *B. yunnanensis* was assembled and analyzed, which will facilitate future research on the evolution of orchid plants and the phylogenetic of the *Bulleyia*.

The leaf samples of *B. yunnanensis* were collected from the Wild Orchid Conservation Center of Yunnan Fengchunfang Biotechnology in Fumin County, Yunnan Province, China (25°20 ′19″ N, 102°27′26″ E). The specimen was deposited in the Herbarium of Southwest Forestry University (HSFU, Lilu 20180005). The total genomic DNA was extracted from the fresh leaf using the modified CTAB procedure of Doyle and Doyle (1987) and sequenced on Illumina Hiseq 2500 platform (Illumina, San Diego, CA) in Shanghai Personal Biotechnology Co., Ltd. With the chloroplast genome of *Pleione formosana* Hayata (GenBank accession number NC_042197) as the reference sequence, we assembled the cp genome from the clean reads by the GetOrganelle pipe-line (Jin et al. 2018), then annotated the new sequences by using the Geneious Prime version 2020.0.4 (Kearse et al. 2012). Finally, the complete cp genome sequence was submitted to the GenBank with accession number MT610368.

The complete chloroplast genome of *B. yunnanensis* was 159,581 bp in length and contained two inverted repeats (IR, 26,652 bp) regions, a large single-copy region (LSC, 87,563 bp), a small single-copy region (SSC, 18,714 bp). This genome sequence encoded 132 genes, including 113 unique genes (79 protein-coding genes, 30 tRNA genes, and four rRNA genes). Among them, the GC content of LSC, SSC and IR regions reached 35.2%, 30.4% and 43.3%, respectively. Besides, GC-content was 37.4% of the overall.

To confirm the phylogenetic position of *B. yunnanensis*, a maximum-likelihood (ML) tree was constructed based on 78 protein-coding genes of nine species from four related tribes in subfamily Epidendroideae as ingroup, with two species from subfamily Orchidoideae as outgroup. These four tribes were selected according to the updated classification of Orchidaceae (Chase et al. 2015; Li et al. 2016) including Cymbidieae Sw., Collabiinae Schltr., Arethuseae Lindl. and Neottieae Lindl. The phylogenetic tree was conducted using RAxML v8.1.11 (Stamatakis et al. 2008; Stamatakis 2014) as implemented on the Cyberinfrastructure for Phylogenetic Research (CIPRES) Science Gateway (http://www.phylo.org/, Miller et al. 2010). Other parameters used the default settings. It was revealed that *B. yunnanensis* was closely related to *Pholidota* in tribe Arethuseae of Epidendroideae (Figure 1).

**Figure 1.**
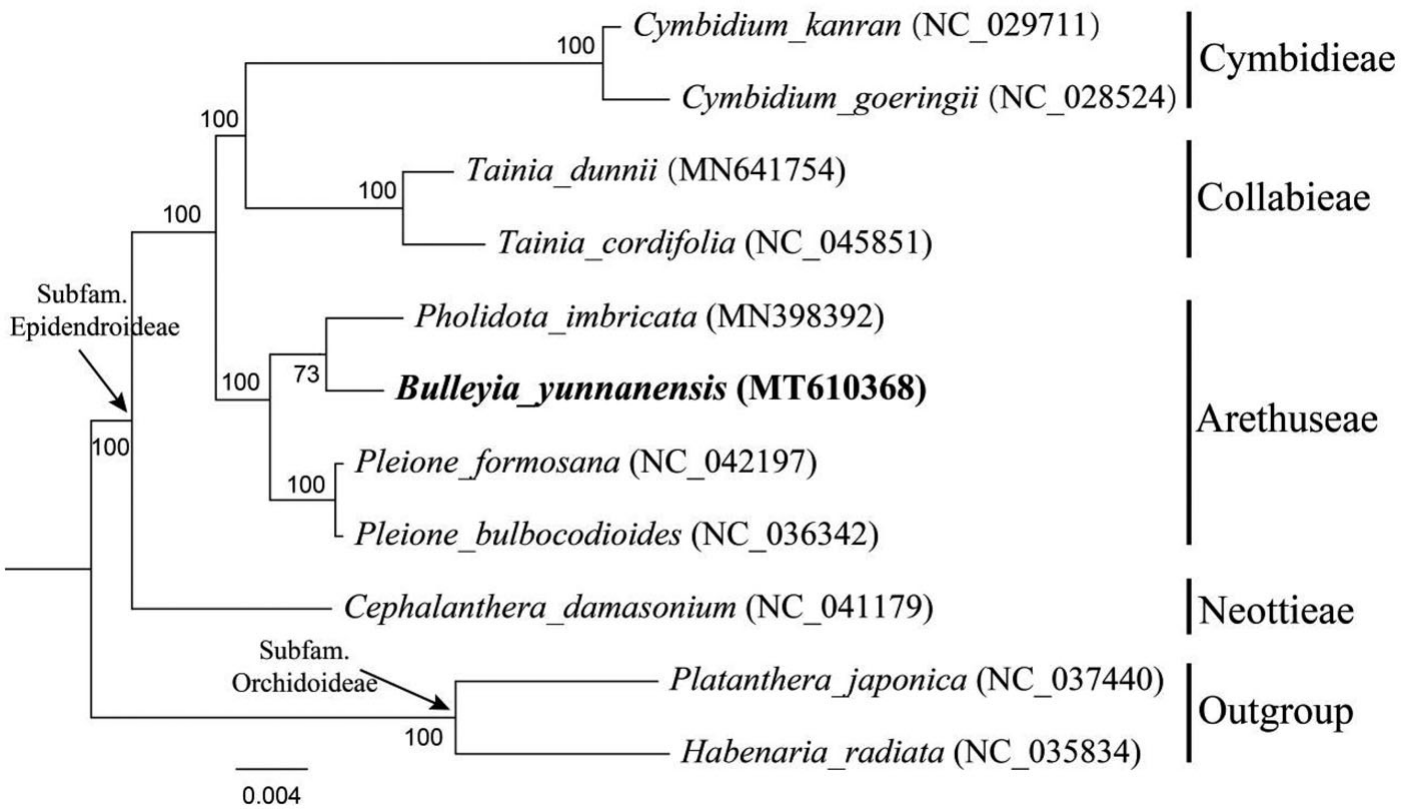
Phylogenetic position of *Bulleyia yunnanensis* inferred by maximum likelihood (ML) based on 78 protein-coding genes of nine species from dour related tribes in subfamily Epidendroideae as ingroup, with two species from subfamily Orchidoideae as outgroup. Sequences used in this study were downloaded from the NCBI GenBank database. The bootstrap values were shown next to the nodes.

## Data Availability

The genome sequence data that support the finding of this study are openly availabie in GenBank of BCBI at https://www.ncbi.nlm,nih.gov under the accession no. MT610368.The associated BioProject, SRA, and Bio-Samplenumbers are PRJN664314,SAMN1625754 and SPX9148609 respectively.
